# Variability in International Society on Thrombosis and Haemostasis-Scientific and Standardization Committee endorsed Bleeding Assessment Tool (ISTH-BAT) score with normal aging in healthy females: contributory factors and clinical significance

**DOI:** 10.1016/j.jtha.2022.11.045

**Published:** 2022-12-27

**Authors:** Dearbhla Doherty, Julie Grabell, Pamela A. Christopherson, Robert R. Montgomery, Barry S. Coller, Michelle Lavin, James S. O’Donnell, Paula D. James, Zimmerman Program Investigators

**Affiliations:** 1 National Coagulation Centre, St James’s Hospital, Dublin, Ireland; 2 Irish Centre for Vascular Biology, School of Pharmacy and Biomolecular Sciences, Royal College of Surgeons in Ireland, Dublin, Ireland; 3 Department of Medicine, Queen’s University, Kingston, Ontario, Canada; 4 Blood Research Institute, Versiti, Milwaukee, WI, USA; 5 Children’s Research Institute, Medical College of Wisconsin, Milwaukee, WI, USA; 6 Allen and Frances Adler Laboratory of Blood and Vascular Biology, Rockefeller University, NY, USA; 7 National Children’s Research Centre, Our Lady’s Children’s Hospital Crumlin, Dublin, Ireland

**Keywords:** blood coagulation disorders, inherited, decision support techniques, hemorrhage, menorrhagia, von Willebrand diseases

## Abstract

**Background:**

Bleeding assessment tools are key screening tests used in the evaluation of patients with suspected inherited bleeding disorders. The International Society on Thrombosis and Haemostasis-Scientific and Standardization Committee endorsed Bleeding Assessment Tool (ISTH-BAT) has differing reference ranges for adult males (0–3), adult females (0–5), and children (0–2), reflecting differing bleeding symptoms and exposure to hemostatic challenges in these healthy population subgroups. Age is known to markedly impact bleeding score in individuals with von Willebrand disease. However, the influence of age on bleeding score in healthy adult controls is poorly understood.

**Objectives:**

We aimed to assess variability in ISTH-BAT score with age among healthy control females.

**Methods:**

We used the legacy “Merging Project” dataset of normal healthy controls upon which current ISTH-BAT normal ranges are based. We included women, totaling 646 individuals. The normal range (middle 95th percentile) of total ISTH-BAT and grouped subdomain scores between age quartiles was assessed.

**Results:**

The normal range of ISTH-BAT scores increased with age, ranging from 0 to 4 in the youngest quartile (age range, 18–30) to 0 to 6 in the oldest (age range, 52–88). This increased variability with aging was related both to high menorrhagia domain scores in older women and an increase in postprocedural bleeding with accumulated exposure to hemostatic challenges.

**Conclusions:**

Cumulatively, our data highlight that normal aging leads to increased variability in bleeding scores in healthy adult females. Further refinement of the ISTH-BAT with age-adjusted reference ranges may improve the sensitivity and specificity of the tool among females.

## 1 | INTRODUCTION

Identification and quantification of abnormal bleeding is a critical step in the diagnostic pathway of inherited bleeding disorders. Indeed, a bleeding phenotype is a prerequisite for some diagnoses, such as the Bleeding Disorder of Unknown Cause and type 1 von Willebrand disease (VWD) with von Willebrand factor (VWF) levels between 30 and 50 International Units/dL. However, reliably differentiating pathologic mucocutaneous bleeding symptoms from the mild hemorrhagic symptoms seen in the general population remains challenging [1–4]. Over the last 20 years, the development and refinement of standardized Bleeding Assessment Tools (BATs) has sought to address this challenge, beginning with the “Vicenza” score in 2005 [5]. Further iterations, primarily focusing on reducing the time of administration, have since been developed [1,6]: most recently with an International Society on Thrombosis and Haemostasis-Scientific and Standardization Committee endorsed Bleeding Assessment Tool (ISTH-BAT) for use in both adult and pediatric settings [7]. Recent international guidelines advocate the use of standardized BATs in screening for VWD in primary care, with the upper limit of the normal range effectively acting as a medical decision limit to trigger investigation and tertiary referral. In addition, standardized BATs are advised in the tertiary referral setting to assess and document the severity of bleeding symptoms, in conjunction with blood testing as part of the initial diagnostic workup [8]. ISTH-BAT scores have also been validated in the setting of inherited platelet disorders [9–11].

A normal range for the ISTH-BAT was established in 2014 using the middle 95th percentile of a large population of normal healthy control individuals [12]. Because of the differences in ISTH-BAT distributions between males and females (related to the sex-specific domains of menorrhagia and postpartum hemorrhage), individual reference ranges exist for males (ISTH-BAT; normal range, 0–3) and females (normal range, 0–5). In addition, a pediatric reference range for individuals <18 years was established (0–2), reflecting the limited number of hemostatic challenges encountered by this population [12,13]. More recently, adolescent-appropriate cut-off score values have been proposed [14].

Importantly, however, the accumulation of hemostatic challenges does not plateau at the age of 18. Indeed, aging has been demonstrated to have an impact on BAT scores in patients with VWD, with BAT score increasing by 1.1 unit per decade in females with moderate to severe VWD in the von Willebrand in the Netherlands (WiN) study [15]. Furthermore, the diagnostic accuracy of bleeding scores in VWD has been shown to be age-dependent, even among adult patients, with Tosetto et al. [16] reporting an age-related increase in the performance of two validated bleeding scores among individuals with VWD. In contrast, the impact of aging on BAT scores in a normal healthy population remains poorly defined [12,17].

We sought to evaluate the impact of aging on ISTH-BAT scores in a large healthy control population of women and to assess the effects of increasing age on specific BAT domains.

## 2 | METHODS

We used the legacy dataset of bleeding scores and demographic data collected from a large cohort of North American healthy control subjects that was used to define the current normal ranges of the ISTH-BAT [12,18]. Demographic data were collected on self-reported age, sex, ethnicity, medical conditions, and medications. Individuals taking regular antiplatelet or anticoagulant medications were excluded from analysis. Owing to the limited numbers of males (n=335) and children (n=328, [169 female, 159 male]) within the cohort, only females ≥18 years old (n=646) were included.

Individuals were grouped by age quartile (Q1, youngest quartile, to Q4, oldest quartile). ISTH-BAT scores were assessed using total score and sub grouped domain scores as follows: “sex-specific domains score” (sum of the menorrhagia and postpartum hemorrhage [PPH] domains) [12], “challenge domains score” (sum of the surgery and dental extraction domains) [11], and “spontaneous domains score” (sum of all other domains). For clarity, the term “menorrhagia domain” is used to describe the ISTH-BAT domain, whereas the International Federation of Gynecology and Obstetrics (FIGO) recommended term “heavy menstrual bleeding” (HMB) is used in descriptive text [7,19].

Statistical analyses were performed in GraphPad Prism 8.4.3 (GraphPad Software, USA) with a P value <0.05 considered statistically significant. A non-parametric method was used to identify percentile ranking, eg, 97.5th percentile = (n + 1) × 0.975. The normal range was taken as the 2.5th to 97.5th percentile of normal individuals, as previously reported [12,20]. Fisher exact test was used to test differences in proportions, whereas logistic regression was used to analyze the relationship between independent variables and between quartiles, reported as odds ratio (OR) with 95% CIs.

## 3 | RESULTS AND DISCUSSION

**FIGURE 1:**
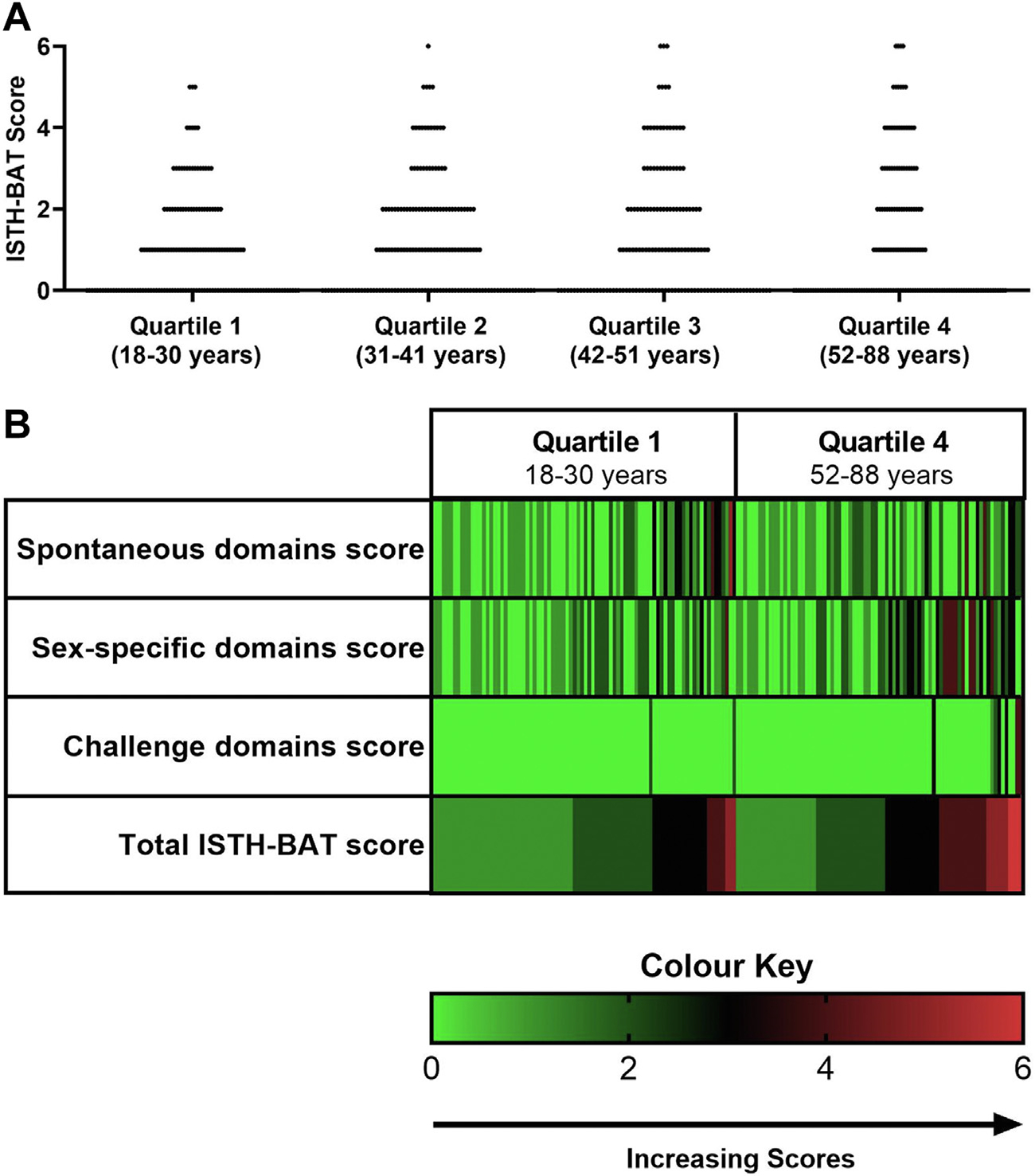
(A) ISTH-BAT score by age quartile among females (Quartile 1-Quartile 4), with each point representing the total ISTH-BAT score of a healthy control individual. Distribution of ISTH-BAT scores changed with normal aging; subjects in the oldest quartile (Q4) were approximately 3 times more likely to have an ISTH-BAT score of ≥4, when compared with those in the youngest quartile (Q1), (Q4 vs Q1; 13.77% vs 4.88%; odds ratio, 3.12; 95% CI, 1.40–6.74). (B) Heat-map visualization illustrating total ISTH-BAT and sub group domain scores (rows) reported by each subject (columns) in the youngest (Q1) and oldest quartile (Q4). Increasing scores are illustrated by color change, from green (score of 0) to red (score of 6), as shown in color key. Individuals in both quartiles had similar distribution of Spontaneous domains scores. In contrast, the distribution of sex-specific domains scores (sum of the Menorrhagia and PPH domains) and challenge domains scores (sum of the Surgery and Dental Extraction domains) broadened with age. Only subjects with ISTH-BAT ≥1 are included for illustrative purposes. ISTH-BAT, International Society on Thrombosis and Haemostasis-Scientific and Standardization Committee endorsed Bleeding Assessment Tool; PPH, postpartum hemorrhage

In this study, 646 females were included, ranging in age from 18 to 88 years old. Age quartile breakdown and demographic data are illustrated in Table 1. For the entire cohort, the normal range for the ISTH-BAT score was 0 to 5, as previously described [12]. However, variability in bleeding scores was observed, with high scores associated with advancing age (Figure 1A, B and Table 1). For example, we observed that subjects in the oldest quartile were approximately three times more likely to have an ISTH-BAT score of ≥4 when compared with those in the youngest quartile (Q4 vs Q1; OR, 3.12; 95% CI, 1.40–6.74) (Figure 1A, B). Importantly, this age-dependent effect influenced the normal range (middle 95th percentile distribution). In particular, when assessed by age quartiles, the normal ranges were shown to be 0 to 4 (Q1, 18–30 years), 0 to 5 (Q2 and Q3, 31–41 years and 42–51 years, respectively), and 0 to 6 (Q4, 52–88 years) (Table 1). Based on these data, applying the current upper limit of the normal range (0–5) as a cut-off may result in under-referral of younger women with abnormal bleeding. Conversely, older women with bleeding within the normal range for their age (score of 6) may be misclassified as abnormal and consequently undergo unnecessary investigation and management.

We next evaluated the domains within the ISTH-BAT score that led to the increased variability with aging. First, we evaluated the “spontaneous domains score.” A similar distribution of spontaneous domains scores was seen between Q1 and Q4 (Figure 1B), with 95% of scores falling within the range between 0 and 3, regardless of age (Table 1). Furthermore, increasing age had no effect on the odds of an individual reporting bleeding in a spontaneous domain (OR, 0.99; 95% CI, 0.98–1.01 for spontaneous domain score >0). This suggests that the increased variability in bleeding scores seen with aging is not due to the accumulation of spontaneous symptoms.

In contrast, age clearly influenced the “sex-specific domains,” with an increase in the normal range seen in older women (Q1[0–2] vs Q4 [0–4]) (Table 1 and Figure 1B). Although rates of PPH were numerically higher in the oldest than in the youngest quartile, this was not statistically significant (6.6% vs 3.1%, *p* = 0.20). In contrast, menorrhagia scores varied markedly with aging, with older women approximately nine times more likely to score ≥3 in the menorrhagia domain (Q4 vs Q1; OR, 9.18; 95% CI, 2.25–40.52). Management of menorrhagia typically follows a step-wise approach, with medical management prioritized in women who choose to conserve their fertility. Surgical management, in contrast, is typically offered to women who fail, are refractory, or are intolerant of medical treatments [21,22]. Importantly, equal rates of older and younger women sought medical attention for HMB (menorrhagia domain score of 1+, Q1 vs Q4; 27.44% vs 28.14%, *p* = 0.90), suggesting that the higher menorrhagia domain scores seen in older women are likely due to the accumulation of treatment options with time, rather than increasing prevalence of HMB with aging. Thus, the menorrhagia domain of the ISTH-BAT, although highly sensitive in the detection of HMB in women with bleeding disorders [23], appears to be implicitly weighted toward older women, even among healthy controls.

Challenge domains were also impacted by age. Although the upper limit of the normal range for postchallenge bleeding was 0 in the youngest age quartile, this increased to 3 in the oldest quartile (Table 1, Figure 1B). Indeed, the odds of reporting a history of postchallenge bleeding increased by 3.3% per year of age (OR, 1.03; 95% CI, 1.003–1.06). Rates of surgical and dental challenges significantly increased with age, with individuals in the oldest quartile 3.8 and 2.6 times more likely to have undergone surgery (Q4 vs Q1; OR, 3.8; 95% CI, 2.30–6.34) and dental extraction, (Q4 vs Q1; OR, 2.6; 95% CI, 1.59–4.42), respectively, when compared with those in the youngest quartile.

**FIGURE 2:**
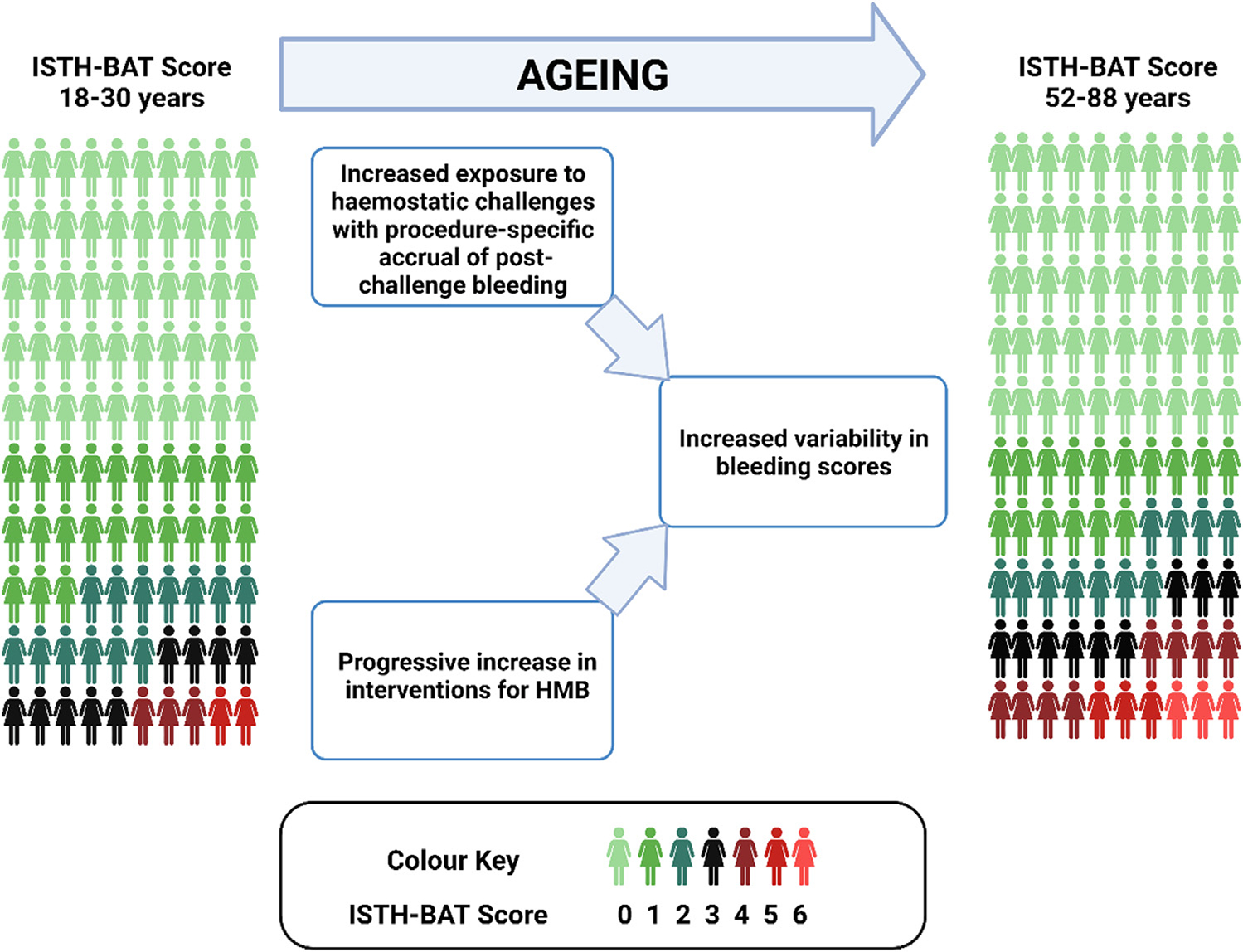
Illustrative representation of variability in ISTH-BAT score with normal aging, Increasing scores are illustrated by color change, from green (score of 0) to red (score of 6), as shown in color key. HMB, heavy menstrual bleeding; ISTH-BAT, International Society on Thrombosis and Haemostasis-Scientific and Standardization Committee endorsed Bleeding Assessment Tool

Interestingly, there was no increased likelihood of postchallenge bleeding in individuals with a history of spontaneous bleeding (OR, 1.647; 95% CI, 0.6039–4.166). In addition, no individual reported a history of bleeding after both dental extraction and surgery. This suggests, as expected in a control population with no known bleeding disorders, that post challenge bleeding occurs because of local or surgical factors, independent of the bleeding history. Thus, our data suggest that the increased variability in bleeding scores seen with aging is in part due to the procedure-specific accumulation of post challenge bleeding as more individuals are exposed to procedures (Figure 2).

Our study has a number of limitations. In the preliminary analysis, we observed a similar increase in rates of surgical and dental challenges with age among healthy control males. However, our dataset was underpowered to evaluate whether a corresponding broadening of bleeding score was also present with aging in males. Owing to the narrow reference range and without the impact of age-associated variation in the menorrhagia domain seen in females, we hypothesize that any age-related effects on ISTH-BAT scores in males is likely to be more subtle and will require large adequately powered cohorts to be detected. Furthermore, although our dataset included women up to the age of 88, only 6% (40/646) of the cohort were above 65 years of age. Further dedicated studies evaluating an older population may reveal an even higher spread of bleeding scores in older healthy females. Finally, as we only included females aged ≥18 years in our analysis, the critical issue of adolescent-specific normal ranges remains to be addressed.

Our data provide the first comprehensive study on the variability of bleeding scores with normal aging and the assessment of factors that drive this variability. Using the large dataset of healthy control individuals upon which the current ISTH-BAT female reference range is based further strengthens our study.

## 4 | CONCLUSION

Cumulatively, our data suggest that normal aging leads to increased variability in bleeding scores among healthy women, related both to step-wise increases in the menorrhagia domain scores among women with HMB and the accrual of postprocedural bleeding with increasing exposure to procedures (Figure 2). We anticipate that if our data are validated by others, further refinement of the ISTH-BAT with age-adjusted reference ranges may improve its sensitivity as a diagnostic tool in younger women, while enhancing specificity in older women. Further studies are required to address the outstanding critical issue of the ISTH-BAT normal range in adolescents and to investigate for the presence of any effect of aging on the distribution of bleeding scores in males.

## Supplementary Material

Appendix

## Figures and Tables

**TABLE 1:** Demographic data and ISTH-BAT data in total female cohort and by age quartile.

		By age quartile			
	Total	Q1	Q2	Q3	Q4

**Demographic data**					
**n**	646	164	165	150	167
**Age, y**	41 (18–88)	25 (18–30)	36 (31–41)	46 (42–51)	59 (52–88)
Median (range)					
**Ethnicity, n (%)** ^a^	n = 634	n = 161	n = 164	n = 146	n = 163
African American	86 (13.6%)	23 (14.3%)	28 (17.1%)	27 (18.5%)	8 (4.9%)
Asian	31 (4.9%)	10 (6.2%)	10 (6.1%)	9 (6.2%)	2 (1.2%)
Other^b^	7 (1.1%)	3 (1.9%)	3 (1.8%)	1 (0.7%)	0 (0%)
White	510 (80.4%)	125 (77.6%)	123 (75.0%)	109 (74.7%)	153 (93.9%)
**ISTH-BAT data**					
**Overall ISTH-BAT score** 2.5th-97.5th percentile	0–5	0–4	0–5	0–5	0–6
**Spontaneous domains score** 2.5th-97.5th percentile	0–3	0–3	0–3	0–3	0–3
**Sex-specific domains score** 2.5th-97.5th percentile	0–4	0–2	0–4	0–4	0–4
**Challenge domains score** 2.5th-97.5th percentile	0–2	0–0	0–2	0–2	0–3

ISTH-BAT, International Society on Thrombosis and Haemostasis-Scientific and Standardization Committee endorsed Bleeding Assessment Tool. Sex-specific domains score (menorrhagia and postpartum hemorrhage [PPH] domains), challenge domains score (surgery and dental extraction domains) and spontaneous domains score (all other domains).

a Denominator is number of individuals with ethnicity data reported.

b Other, including American Indian, Pacific Islander and multiracial.

## Essentials:

- Bleeding scores, such as the International Society on Thrombosis and Haemostasis-Scientific and Standardization Committee endorsed Bleeding Assessment Tool (ISTH-BAT), are used in the investigation of bleeding disorders.
- Different normal ranges of the ISTH-BAT currently exist for males, females, and children.
- Our data highlight that ISTH-BAT normal ranges vary with age in adult female healthy controls.
- This is because of higher menorrhagia scores and accrual of post challenge bleeding in older women.
