# Supplementary material for: Variability in International Society on Thrombosis and Haemostasis-Scientific and Standardization Committee endorsed Bleeding Assessment Tool (ISTH-BAT) score with normal aging in healthy females: contributory factors and clinical significance
Source: J Thromb Haemost. Author manuscript; Available in PMC 2024 Apr 11. (PMC11008594; doi:10.1016/j.jtha.2022.11.045)
Supplement: Appendix [file NIHMS1980403-supplement-Appendix.docx]

**Appendix:**

**Zimmerman Program Investigators:**

**Principal Investigators include** R. Montgomery, V. Flood, S. Haberichter, T. Abshire, H. Weiler, Versiti Blood Research Institute, Milwaukee, WI; D. Lillicrap, P. James, Queen’s University, Kingston, ON, Canada; J. O’Donnell, Royal College of Surgeons in Ireland, Dublin, Ireland, C. Ng, University of Colorado, Denver, CO; J. Di Paola, B. Sadler, Washington University in St. Louis, St. Louis, MO.

**Directors  of  the  primary  centers  include**  T. Abshire, C. Bennett, R. Sidonio, Emory University School of Medicine, Atlanta, GA; M. Manco-Johnson, J. Di Paola, C. Ng, Mountain States Regional Hemophilia and Thrombosis Center, Aurora, CO; J. Journeycake, A. Zia, UT Southwestern, Dallas, TX; J. Lusher, M. Rajpurkar, Wayne State University, Detroit, MI; A. Shapiro, Indiana Hemophilia & Thrombosis Center, Indianapolis, IN; S. Lentz, University of Iowa, Iowa City, IA; J. Gill, V. Flood, Comprehensive Center for Bleeding Disorders, Milwaukee, WI; C. Leissinger, Tulane University Health Sciences Center, New Orleans, LA; M. Ragni, University of Pittsburgh, Pittsburgh, PA; M. Tarantino, J. Roberts, Bleeding & Clotting Disorders Institute, Peoria, IL; P. James, Queen’s University, Kingston, ON, Canada.
